# Has the consistency evaluation policy of generic drugs promoted the innovation quality of Chinese pharmaceutical manufacturing industry? An empirical study based on the difference-in-differences model

**DOI:** 10.3389/fpubh.2023.1265756

**Published:** 2023-12-01

**Authors:** Qiang Liu, Zhe Huang, Zhenbin Mao

**Affiliations:** ^1^School of Business Administration, Shenyang Pharmaceutical University, Shenyang, China; ^2^Research Institute of Drug Regulatory Science, Shenyang Pharmaceutical University, Shenyang, China

**Keywords:** consistent evaluation policy of generic drugs, innovation quality, difference-in-differences model, pharmaceutical manufacturing industry, mediating effect

## Abstract

**Introduction:**

In March 2016, the Chinese government officially launched a nationwide consistency evaluation of the quality and efficacy of generic drugs.

**Methods:**

This paper conducted an empirical study using the Difference-in-Differences method to explore the effect of this policy on the innovation quality of China’s pharmaceutical manufacturing industry and further analyzed the underlying mechanism of action.

**Results:**

The results of the study show that the generic consistency evaluation policy has a significant promotion effect on the innovation quality of China’s pharmaceutical manufacturing industry, and the promotion effect is the largest for non-state-owned enterprises and enterprises in the central region; in addition, the intensity of R&D capital investment and R&D personnel investment which play a mediating role.

**Discussion:**

Therefore, we should fully recognize the positive effect of generic drug consistency evaluation policy on improving the innovation quality of the pharmaceutical manufacturing industry and pay attention to the necessity of regional coordination and unification in policy implementation and the formulation of supporting policy tools. This study provides empirical evidence for the implementation effect of the generic drug consistency evaluation policy, which can provide an essential reference for the further improvement of the procedure and the R&D decision-making of pharmaceutical enterprises.

## 1 Introduction

To improve the quality of generic drug products, promote the high-level development of the entire pharmaceutical manufacturing industry, and guarantee the safety and effectiveness of people’s medicines, all major pharmaceutical countries have carried out consistent evaluations of generic drugs. In China, on January 20, 2012, the State Council issued the “Twelfth Five-Year Plan for National Drug Safety”, which, for the first time, put forward the requirement to carry out the consistent evaluation of generic drugs. In March 2016, the General Office of the State Council formally announced the “Opinions on Carrying Out the Consistency Evaluation of the Quality and Efficacy of Generic Drugs” [(1) No. 8]. In 2020, the “Measures for the Administration of Pharmaceutical Registration” explicitly put forward the requirements of bioequivalence testing, which requires that the generic drug must be consistent with the reference preparation in terms of quality and clinical efficacy (2). Consistency evaluation of generic drugs is a historical remedial lesson in regulating generic drugs in China and a reference for the law of drugs in Japan and the United States (3). To this end, the State Drug Administration has established and improved the working mechanism of generic drug consistency evaluation and expressly set up the Office of Consistency Evaluation of Generic Drug Quality and Efficacy to coordinate and promote the work. An expert committee composed of more than 70 well-known experts from the pharmaceutical industry, associations, societies, colleges, and universities has been formed to convene specialist consultation meetings on critical technical issues such as the selection of reference preparations, exemptions from human bioequivalence tests, and evaluation programs for complex varieties, to strengthen the technical support for consistency evaluation and guarantee the fairness and scientificity of the evaluation results (4). As of July 2023, the Center for Drug Evaluation (CDE) of the State Drug Administration has issued more than 50 technical guidelines and 70 batches of catalogs of reference preparations (4) on the consistency evaluation of generic drugs. Both pharmaceutical regulatory authorities and pharmaceutical enterprises or research institutions in China have invested much experience in generic drug consistency evaluation. As of December 31, 2022, China has passed the consistency evaluation (including deemed over-evaluation) of 5,573 acceptance numbers involving 4,013 drug specifications (5). While high-quality development of generic drugs is significant, we should also pay attention to the development of originator innovator drugs. At present, many scholars have theoretically analyzed the relationship between the consistent evaluation policy for generic medicines and the quality of innovation in the pharmaceutical manufacturing industry, but there are fewer empirical studies.

Based on the relevant data of 231 A-share pharmaceutical manufacturing listed companies from 2013–2021 as the research basis, this paper conducted an empirical study on this issue. The study found that the generic consistency evaluation policy has a significant role in promoting the quality of innovation in the pharmaceutical manufacturing industry. The possible contributions of this paper are:

1. The relationship between generic consistency evaluation and innovation quality of the pharmaceutical manufacturing industry is empirically studied for the first time, which provides a more strategic value for promoting the consistency evaluation of generic drugs in China.
2. It enriches the research results of the policy of consistent evaluation of generic drugs from the empirical research perspective.
3. After analyzing the overall impact effects, it conducts a sub-regional and enterprise heterogeneity analysis and further explores the impact mechanism of generic drug consistency evaluation policy on innovation quality.

## 2 Institutional background and literature review

A generic drug is a drug that has the same active ingredients, dosage form, specifications, route of administration, indications, and other characteristics as the reference original drug and is bioequivalent to the reference original drug (6). Generic drugs dominate the world’s pharmaceutical market and have become the basis for medical and livelihood medicines in countries worldwide. According to statistics, in the United States, there are more than 3,000 drug manufacturers, of which more than 2,900 are manufacturers of generic drugs, more than 95% of the medicines declared by enterprises are also generic drugs, and more than 95% of the drugs used in medical institutions are also generic drugs (7). The safety and efficacy of generic medicines will directly affect the clinical therapeutic effect of patients and even the strategic security of a country and national health. However, due to historical, technical, and conceptual reasons, there is no mandatory requirement for consistent evaluation of the clinical efficacy of generic drugs approved for marketing in the past with that of the original drugs, resulting in a particular gap between the quality and effectiveness of certain generic drugs and the original drugs.

Especially after the incident of the “Thalidomide scandal, “the U.S. FDA gradually realized the importance and urgency of consistent evaluation of the quality and efficacy of generic drugs; therefore, the Drug Law Amendments (also known as as the “Kefauver-Harris Amendments”) issued by the FDA in 1962, it has started the Drug Efficacy Study Implementation (DESI) program to demonstrate that all drugs should be safe and effective. The program aims to retrospectively review historical safety gaps due to inadequate drug review regulations and to bring generic drugs into compliance with drug safety and efficacy standards. The program has upgraded the generic drug consistency evaluation from superficial “chemical composition similarity” to “bioequivalence”, eliminating about 6,000 unqualified drugs (8, 9). Similarly, the Ministry of Health and Welfare of Japan clearly states in the “Re-examination and Re-evaluation System” that “with the development of the pharmaceutical industry and the continuous enrichment of pharmaceutical knowledge, the system of re-evaluation of licensed pharmaceuticals based on the current level of pharmacy is called the Pharmaceutical Re-evaluation System, “and three large-scale re-evaluations of pharmaceuticals has been carried out since 1971, mainly consisting of efficacy re-evaluation and quality re-evaluation (10, 11). In April 1980, the Pharmaceutical Affairs Law of Japan incorporated the drug re-evaluation system. By 2001, the re-evaluation of 1,362 preparations of 706 varieties had been completed, and the number of manufacturing enterprises had been reduced and integrated from more than 1,300 to more than 100 (12, 13). The practice of the world’s pharmaceutical powerhouses has proved that the evaluation of the efficacy and quality consistency of generic drugs with the originator drugs has deepened the comparative study of the pharmacodynamics of the generic drugs and the originator drugs by the enterprises, promoted the in-depth analysis of the production process and prescription by the local enterprises, drastically cut down the number of the low-end generic medicines, improved the quality of generic drugs, and ultimately facilitated the development and enhancement of the entire pharmaceutical industry chain (14).

Like developed countries, generic drug strategy is an essential part of China’s drug safety strategy, and the World Bank pointed out in “Generic Drug Policy – The Cornerstone of China’s Essential Drug Policy” published in 2010, that generic drug policy is an indispensable part of China’s essential drug policy (15). China is a large country of generic drug production, with more than 5,000 generic drug manufacturers (16); however, it is not a strong country of generic drugs, and although the number of domestically produced generic drug enterprises is huge, the overall quality is not high. According to each enterprise’s public financial report data, none of the Chinese enterprises will be on the list of the global generic drug TOP10 pharmaceutical enterprises in 2022. To improve the quality of domestic generic drugs, China also officially started to implement generic drug consistency evaluation in 2016.

Although the use of generic drugs in China is enormous, and the implementation of the consistency evaluation policy further promotes the healthy and orderly development of the generic drug market, there are still challenges to the research and development of innovative drugs. There is no contradiction between imitation and innovation, and the development of high-quality generic drugs, especially first-generation drugs, is itself a systematic innovation process.

Therefore, with the in-depth promotion of generic drug consistency evaluation, the future market pattern of China’s pharmaceutical manufacturing industry will move towards the development direction of high-end generic drugs and original, innovative drugs (17, 18). The Chinese government has put forward the goal of “promoting high-quality economic development” (19) and pointed out that innovation is the most important driving force to lead economic development. Therefore, high-quality economic growth must connect to high-quality innovation activities, and high-quality innovation is also a critical factor in promoting the high-quality development of the pharmaceutical manufacturing industry. While the high-quality development of generic drugs is essential, developing original, innovative medicines should be addressed. The pharmaceutical manufacturing industry is not only a technological innovation-driven industry integrating multiple advanced technologies, knowledge of multiple disciplines, and multiple upstream and downstream industrial resources but also a policy innovation-dependent industry highly dependent on changes in national regulatory policies. Therefore, innovation will be the key element throughout the industrial upgrading of China’s pharmaceutical manufacturing industry. As China’s national economic level continues to improve, people’s awareness of health care increases yearly, and the pharmaceutical manufacturing industry for industrial upgrading enhances the quality of innovation needs to become increasingly urgent.

In the existing related studies, some scholars have theoretically proposed that the generic consistency evaluation policy can promote pharmaceutical enterprises to increase R&D investment (18, 20, 21), and some scholars have suggested that the implementation of generic consistency evaluation can encourage the transformation of China’s pharmaceutical industry from imitation to innovation (22, 23). However, there needs to be more relevant research on whether this policy improves the overall innovation quality of China’s pharmaceutical manufacturing industry. Therefore, this paper regards the generic consistency evaluation policy, which was formally promoted and implemented in China in 2016, as a quasi-natural experiment and adopts the DID method to study this viewpoint to test the natural impact effect of the generic consistency evaluation policy on the innovation quality of the pharmaceutical manufacturing industry.

## 3 Theoretical foundation and research hypothesis

The early production process of generic drug products in China mainly imitated the production standards of the original drugs rather than replicating the core efficacy, which led to a significant difference between the prescription, process, and even efficacy of generic and original drugs. Implementing the generic drug consistency evaluation policy will promote the formation of higher production and registration approval standards for generic drugs in China, which will, in turn, force the entire pharmaceutical manufacturing industry to improve production quality. For example, oral solid preparations in the past mainly used *in vitro* dissolution curves as the quality evaluation method, but the technique does not represent clinical equivalence. Therefore, the former CFDA’s announcement of “Opinions on Conducting Consistency Evaluation of the Quality and Efficacy of Generic Drugs” issued in 2016 pointed out that “except for varieties that comply with the principle of exempting bioequivalence tests, drug manufacturers should, in principle, use *in vivo* bioequivalence tests for consistency evaluation.” As a result, the consistency evaluation of the quality and efficacy of generic drugs has upgraded from emphasizing the consistency evaluation of *in vitro* dissolution profiles in the past to the consistency evaluation of bioequivalence (24). The improved evaluation standard will encourage existing generic drug manufacturers to conduct in-depth research on the originator reference drug’s prescription process, active ingredients, and bioequivalence.

In contrast, the originator reference preparation often restricts the marketing of generics through commercial strategies or by setting up patent barriers, such as using multiple drug delivery systems and different polymers and their ratios used in the prescription (25). To circumvent these barriers, generic manufacturers must innovate, especially for first-in-class generic drugs, which cannot use the same formulation and manufacturing process as the original drug, yet must demonstrate bioequivalence in the clinic. From the perspective of intellectual property protection, advantageous human, technological, and material resources will be redistributed in the process of innovation, promoting a reasonable inflow of resource elements into innovative enterprises, thus optimizing the market competitive environment and forcing enterprises to choose open innovation around core competitiveness (26). Therefore, the process of high-quality imitation will strengthen the enterprises’ learning and understanding of the original drug, form a batch of scientific research results with independent intellectual property rights, realize the cultivation of high-end R&D talents and the accumulation of technological experience, and ultimately, it will promote the improvement of the quality of innovation in China’s pharmaceutical manufacturing industry.

Under the background of supply-side reform, the implementation of consistency evaluation will optimize the capacity structure and resource allocation of the pharmaceutical manufacturing industry, eliminating those enterprises that can only carry out low-end and superficial imitation; at the same time, the linkage of consistency evaluation results with policies such as the right of clinical priority use, centralized band purchasing of medicines, and the selection of the essential drug catalog, etc., will make the varieties of varieties that have passed the consistency evaluation of generic drugs and their manufacturers stand out, and the market, production The market, production and R&D resources are gradually concentrated towards the truly powerful enterprises, resulting in the strongest and the survival of the fittest, which in turn promotes the improvement of China’s generic drug industry’s overall innovation ability and innovation quality level; while some enterprises that have lost their competitive advantages in the generic drug market will be forced to take the initiative to transform in order to survive and develop, and to actively carry out independent R&D and technological innovation, in particular to carry out high-quality creations in order to reshape their own competitive advantages. Therefore, whether active or passive, it will ultimately promote the overall quality of innovation in China’s pharmaceutical manufacturing industry.

In addition, consistent evaluation of generic drugs is similar to the development of new medicines, which still requires enterprises to invest large-scale R&D funds and R&D personnel to carry out the proposed research against the reference preparation. According to the sample survey data, if the enterprise adopts the complete outsourcing method to carry out the consistency evaluation work, the current cost of pharmacy research has risen to nearly 2 million yuan, and the cost of clinical trials has soared to about 6 million yuan. The price of the trials will be as high as about 10 million yuan if the clinical effectiveness trials are carried out (20, 27). If each number participating in consistency evaluation is calculated according to the average investment of 5 million yuan (20), China’s existing 95,000 (28) chemical drug approval number if all participate in consistency evaluation, the entire pharmaceutical manufacturing industry needs to invest in research and development funds will be as high as 400 billion yuan or more. In 2021, the primary business income of China’s pharmaceutical manufacturing industry is 2958.2 billion yuan, which estimates that the intensity of R&D capital investment (R&D investment/primary business income) used only to complete the consistency evaluation will reach 16%, close to the R&D investment intensity of developed countries in Europe and the United States or the international pharmaceutical giant enterprises (29). At the same time, the process requirements of high-standard consistency evaluation and the R&D needs of high-level generic drugs, as well as the development direction of independent, innovative drug development, will also enhance the willingness of enterprises to recruit high-end R&D talents. Having sufficient R&D funds and a team of high-end R&D talents will also promote improving the innovation quality of the entire pharmaceutical manufacturing industry.

Finally, according to the viewpoint of signaling theory, the implementation of the policy of “consistency evaluation of generic drugs” will send a signal to the whole market that the state “reduces low-end repetitive imitations and encourages innovation of original drugs”, which in turn will enhance the confidence of enterprises in improving their own R&D and innovation capabilities, and increase the input of innovation factors and resources, thus promoting the innovation behavior of the whole industry in the direction of high-quality innovation.

Based on the above analysis, this paper puts forward the following hypotheses:

*H1*: The generic consistency evaluation policy can improve the innovation quality of China's pharmaceutical manufacturing industry.

*H2*: The generic consistency evaluation policy enhances the innovation quality of China's pharmaceutical manufacturing industry by improving enterprise R&D capital investment intensity.

*H3*: The generic consistency evaluation policy enhances the innovation quality of China's pharmaceutical manufacturing industry by improving the intensity of enterprise R&D labor input.

**Figure 1:**
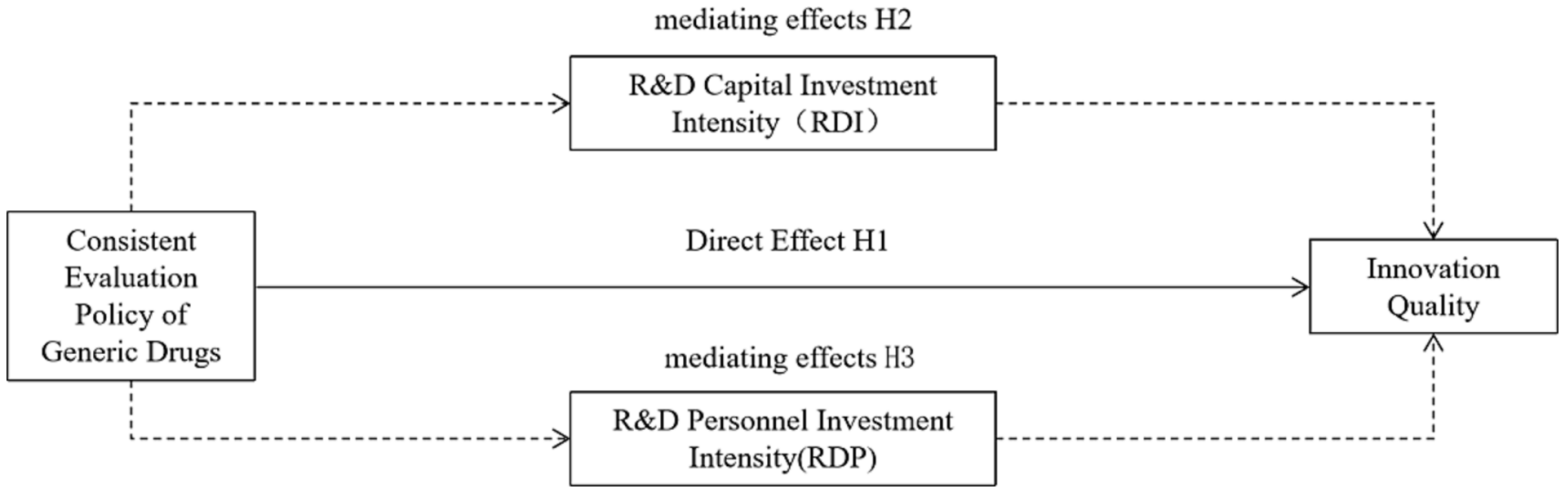
Theoretical framework diagram.

The theoretical hypothesis model of this paper is shown in Figure 1.

## 4 Research design

### 4.1 Data sources and sample selection

This paper’s samples and raw data come from the company research series database of China Stock Market Accounting Research (CSMAR) and the State Intellectual Property Office (SIPO). According to the classification of industry names in the database of CSMAR, the enterprises of the “Pharmaceutical Manufacturing Industry” are selected. The raw data are firstly processed as follows: (1) enterprises with listing status of ST, delisting and consolidation period, suspension and termination of listing are excluded, and retained the samples of enterprises with the normal listing; (2) enterprises with incomplete disclosure of the lack of primary data, indexes, and related data are excluded; (3) remove the sample of companies with abnormal data; (4) remove the sample of companies listed after December 31, 2012; and (5) to reduce the impact of outliers, this paper carries out 1% and 99% tail reduction processing for the continuous variables involved. Finally, the relevant data of 231 A-share pharmaceutical manufacturing listed companies from 2013 to 2021 are selected for empirical research, totaling 1,518 observations.

### 4.2 Variable selection and explanation

#### 4.2.1 Dependent variable: innovation quality

The core explanatory variable of this paper is the innovation quality of the pharmaceutical manufacturing industry. Regarding the innovation quality, most current scholars measure it from the perspective of patents. Lanjouw and Schankerman (30) use the quality of patents to represent the quality of innovation and form an index to describe the quality of patents by using other related indexes, such as the number of forward and backward citations. Zhang et al. (31) used patent grant rate and patent length to measure innovation quality. In this paper, we refer to the studies of Hall et al. (32), Daniel et al. (33), and Meng et al. (34) to measure the innovation quality of enterprises by the number of patents and other citations of listed companies. Drawing on Cao Chunfang’s approach (35), we use the natural logarithm of the average number of patent additional citations filed by firms in the following year plus one, denoted as IQ.

#### 4.2.2 Independent variable

In March 2016, the General Office of the State Council formally announced to the public the Opinions on Carrying Out Consistent Evaluation of the Quality and Efficacy of Generic Drugs [(1) No. 8], which clarified the object and timeframe for participating in the evaluation, the principle of selection of reference preparations, the evaluation methodology and the attribution of responsibility. It formally opened the work of consistency evaluation of generic drugs in China. Therefore, this paper takes 2016 as the time point of policy implementation, and *Post* denotes the time dummy variable, if Post≥2016 ,which is assigned a value of 1 and 0 if Post<2016 .

We determined whether the enterprises have participated in generic drug consistency evaluation by querying the annual reports of enterprises. We set the sample enterprises participating in the generic consistency evaluation as the treatment group; the sample enterprises not attending the generic consistency evaluation are classified as the control group, with Treat denoting the group dummy variable. The enterprises in the treatment group are assigned a value of 1, and the enterprises in the control group are given a value of 0.

Since the explanatory variables are determined by the Post and Treat variables, respectively, this paper sets the explanatory variables as Post and Treat cross-multiplier terms, i.e. $\mathrm{DID}={\mathrm{Treat}}_{i,t}\times {\mathrm{Post}}_{i,t}$ .

#### 4.2.3 Control variables

To reduce the impact of factors other than the core explanatory variables on the explanatory variables, and regarding the research methodology of the existing literature (18, 36–38), this paper selects seven firm-level influences as a combination of control variables, including firm size, capital structure, firm solvency, firm profitability, firm growth, equity concentration, and firm market competitiveness.

#### 4.2.4 Mediating variable

In this paper, the R&D capital investment intensity (RDI) and R&D personnel investment intensity (RDP) of the sample firms are used as mediating variables.

The names, definitions and calculations of all variables in this paper are shown in Table 1. Among them, the data on capital structure (Lev), corporate solvency (Cash), corporate profitability (Roe), corporate growth (Growth), shareholding concentration (Shareh10) and company market competitiveness (Comp) are all from the original data provided by CSMAR; the data of enterprise size (Size), R&D capital investment intensity (RDI) and R&D personnel investment intensity (RDP) are calculated from the original data in the CSMAR.

**Table 1:** Definition of variables.

Variable type	Variable name	Variable symbol	Description
Dependent variable	Innovation Quality	IQ	Ln (average number of citations for patent applications filed by enterprises in the following year +1)
Independent variable	consistent evaluation policy of generic drugs	DID	The cross-multiplier of the grouping dummy variable with the time dummy variable, which takes the value of 0 or 1
Mediating variable	R&D capital investment intensity	RDI	Ratio of enterprise R&D capital investment to operating revenue (%)
	R&D personnel investment intensity	RDP	Number of R&D personnel in enterprises as a percentage (%)
Control variables	Enterprise size	Size	Ln (total enterprise assets)
	Capital structure	Lev	Capital structure
	Corporate solvency	Cash	Cash ratio
	Corporate profitability	Roe	Return on equity
	Enterprise growth capacity	Growth	Company’s growth ability
	shareholding concentration	Shareh10	Ownership concentration(%)
	Market competition	Comp	Industry Lerner index

The descriptive statistics for each variable are shown in Table 2.

**Table 2:** Results of descriptive statistics.

Var name	Obs	Mean	SD	Min	Median	Max
IQ	1,518	0.449	0.306	0.000	0.454	1.299
DID	1,518	0.416	0.493	0.000	0.000	1.000
Treat	1,518	0.582	0.493	0.000	1.000	1.000
Post	1,518	0.739	0.439	0.000	1.000	1.000
Size	1,518	22.071	0.904	20.320	22.010	24.312
Lev	1,518	0.309	0.167	0.046	0.285	0.779
Cash	1,518	1.211	1.521	0.063	0.675	8.815
Roe	1,518	0.086	0.107	−0.466	0.090	0.399
Growth	1,518	−0.400	4.185	−27.659	−0.129	20.058
Shareh10	1,518	57.773	14.606	23.470	58.000	89.350
Comp	1,518	0.168	0.032	0.048	0.168	0.220
RDP	1,518	11.407	8.976	0.000	11.010	41.150
RDI	1,518	5.976	5.620	0.000	4.420	38.320

### 4.3 Equation design

The traditional regression model is challenging to deal with the endogeneity problem caused by omitted variables and other reasons. In contrast, the Difference-in-Differences Model avoids the endogeneity problem to a large extent, helps to study the net effect of the policy, and is one of the more mature empirical methods in policy evaluation. Therefore, to explore the impact of generic drug consistency evaluation policy on the quality of innovation in the pharmaceutical manufacturing industry, this paper constructs a Difference-in-Differences Model to conduct empirical research.

We first conducted the Hausman test and F-test to determine what kind of model is suitable for the panel data used in this paper. The Hausman test can decide whether to choose a random effect model or a fixed effect model, and the F-test can determine whether to select a fixed effect model or a mixed effect model. The test results are shown in Table 3. The test results rejected the original hypothesis; therefore, we used the fixed effect model for regression.

**Table 3:** Results of panel model selection.

	Result
Hausman test	44.184
*p*-value	0.000
*F*-Test	2.13
*p*-value	0.000

Combining the above analysis, the model of this paper is set as follows:


(1) $$IQ_{\mathrm{it}}=\alpha_{0}+\mathrm{\beta Trea}t_{\mathrm{it}}\times Post_{\mathrm{it}}+\mathrm{\theta Contro}l_{\mathrm{it}}+\gamma_{i}+\lambda_{t}+\varepsilon_{\mathrm{it}}$$

where *i* denotes firms and *t* denotes time, $\mathrm{Trea}t_{\mathrm{it}}$ is a group dummy variable, ${\mathrm{Treat}}_{\mathrm{it}}$ takes the value of 1 when the sample firm participates in generic consistency evaluation, and 0 otherwise; ${\mathrm{Post}}_{\mathrm{it}}$ is a time dummy variable; when ${\mathrm{Post}}_{\mathrm{it}}\geq$ 2016, then ${\mathrm{Post}}_{\mathrm{it}}$ =1; when ${\mathrm{Post}}_{\mathrm{it}}$ < 2016, then ${\mathrm{Post}}_{\mathrm{it}}$ = 0; $\mathrm{DID}={\mathrm{Treat}}_{\mathrm{it}}\times {\mathrm{Post}}_{\mathrm{it}}$ ; $\alpha_{0}$ is a constant term, β is the effect of generic consistency evaluation policy on innovation quality, ${\mathrm{Control}}_{\mathrm{it}}$ is the set of control variables, $\gamma_{i}$ represents individual firm fixed effects, $\lambda_{t}$ represents time fixed effects, and $\varepsilon_{\mathrm{it}}$ represents a random perturbation term.

## 5 Empirical results and analysis

### 5.1 Correlation test

To ensure that the empirical analysis has practical significance, this paper first conducted the test of the Pearson correlation coefficient matrix for each variable included in the benchmark regression model, and the test results are shown in Table 4. The results show that the core explanatory variable negatively correlates with IQ, which is inconsistent with the expected hypothesis. Still, considering that the correlation coefficient matrix only measures the relationship between the corresponding bivariate variables and does not exclude the interference of the control variables and latent variables (such as the time effect and the individual effect), the specific correlation relationship needs to be further analyzed by regression analysis to determine.

**Table 4:** Results of correlation test.

	IQ	DID	Treat	Post	Size	Lev	Cash	ROE	Growth	Shareh10	Comp
IQ	1.000										
DID	−0.183***	1.000									
Treat	−0.088***	0.716***	1.000								
Post	−0.238***	0.502***	−0.063**	1.000							
Size	−0.033	0.255***	0.240***	0.133***	1.000						
Lev	−0.083***	0.135***	0.204***	−0.055**	0.251***	1.000					
Cash	0.118***	−0.142***	−0.120***	−0.089***	−0.152***	−0.542***	1.000				
Roe	0.156***	−0.111***	−0.088***	−0.026	0.121***	−0.260***	0.101***	1.000			
Growth	0.097***	−0.041	−0.002	−0.045*	−0.029	−0.092***	0.037	0.319***	1.000		
Shareh10	0.095***	−0.054**	−0.088***	0.035	0.070***	−0.214***	0.219***	0.205***	0.062**	1.000	
Comp	−0.199***	0.249***	−0.104***	0.622***	0.165***	−0.087***	−0.030	0.031	−0.072***	0.094***	1.000

*t*-statistics in parentheses. *** *p* < 0.01, ** *p* < 0.05, * *p* < 0.1.

### 5.2 Tests for covariance

A multicollinearity test is needed to avoid the impact of data covariance on the empirical results. The Variance Inflation Factor (VIF)can detect multicollinearity, which is the ratio of the variance when there is multicollinearity between variables to the variance when there is no multicollinearity; generally, when 0 < VIF < 10, there is no multicollinearity; when 10 ≤ VIF < 100, there is strong multicollinearity; when VIF ≥ 100, there is serious multicollinearity (39). The results of the covariance test are shown in Table 5. The results show that the VIF value of each variable is less than 10, indicating that the indicators selected in this paper do not have covariance.

**Table 5:** Results of covariance test.

	Lev	Cash	Roe	Size	DID	Growth	Comp	Shareh10	Mean VIF
VIF	1.662	1.467	1.287	1.227	1.163	1.127	1.126	1.115	1.272

### 5.3 Baseline empirical results

Combining the results of the previous Hausman and F-test to exclude the endogeneity problem caused by individual effect and time effect, this paper finally adopts the fixed effect model for regression analysis. At the same time, since patent-related innovation quality indicators have a specific time lag relative to the implementation of policies and R&D investment, drawing on Kong Dongmin et al.’s approach (40), this paper treats the independent variables in the regression with first-order lags and second-order lags again, respectively. In addition, to enhance the reliability of the results, stepwise regression is used to verify the hypotheses, and the results are shown in Table 6. In each group of regressions, the core explanatory variable’s estimated coefficients were significantly positive at the 1% statistical level. This indicates that implementing the policy of consistency evaluation of generic drugs has a significant role in promoting innovation quality in China’s pharmaceutical manufacturing industry.

**Table 6:** Baseline empirical results.

	(1)	(2)	(3)	(4)	(5)	(6)	(7)	(8)	1st order lag	2nd order lag
Variables	IQ	IQ	IQ	IQ	IQ	IQ	IQ	IQ	IQ	IQ
DID	0.073*** (7.96)	0.076*** (7.94)	0.077*** (8.07)	0.075*** (8.51)	0.076*** (8.60)	0.076*** (8.67)	0.076*** (8.77)	0.073*** (8.93)	0.059*** (8.77)	
Size		0.032*** (4.37)	0.035*** (5.43)	0.037*** (5.07)	0.032*** (4.17)	0.032*** (4.13)	0.032*** (4.07)	0.029*** (4.04)		0.048*** (7.55)
Lev			−0.059* (−1.77)	0.020 (0.29)	0.044 (0.66)	0.045 (0.69)	0.051 (0.75)	0.041 (0.65)	0.030 (1.45)	0.053*** (2.97)
Cash				0.015* (1.73)	0.016* (1.84)	0.016* (1.85)	0.016* (1.85)	0.016* (1.85)	0.038 (0.51)	0.130* (1.86)
Roe					0.092*** (3.75)	0.073** (2.44)	0.072** (2.39)	0.077*** (2.89)	0.008 (1.38)	0.012* (1.76)
Growth						0.001*** (3.45)	0.001*** (3.43)	0.001*** (4.44)	0.097*** (3.45)	0.076*** (3.10)
Shareh10							0.000 (1.01)	0.001 (1.38)	0.000 (0.44)	0.001 (0.92)
Comp								−1.160** (−2.00)	0.000 (0.45)	0.000 (0.09)
Constant	0.638*** (98.26)	−0.575*** (−3.36)	−0.618*** (−4.01)	−0.706*** (−3.69)	−0.620*** (−3.17)	−0.613*** (−3.12)	−0.632*** (−3.00)	−0.320** (−2.07)	−1.307** (−2.29)	−1.489* (−1.68)
Observations	1,518	1,518	1,518	1,518	1,518	1,518	1,518	1,518	−0.275	−0.768
R-squared	0.334	0.335	0.335	0.338	0.338	0.339	0.339	0.341	(−0.57)	(−1.51)
Number of groups	231	231	231	231	231	231	231	231	1,279	1,065
Company	Yes	Yes	Yes	Yes	Yes	Yes	Yes	Yes	0.367	0.367
Year	Yes	Yes	Yes	Yes	Yes	Yes	Yes	Yes	219	200
									Yes	Yes
									Yes	Yes

*t*-statistics in parentheses. *** *p* < 0.01, ** *p* < 0.05, * *p* < 0.1.

Hypothesis H1 is preliminarily verified.

### 5.4 Parallel trend test

When applying the DID model to test the policy effect, a critical prerequisite assumption that needs to be satisfied is that the explanatory variables meet the parallel trend before the policy occurs, i.e., the innovation quality of the treatment group and the control group have the same trend of change before the implementation of the consistency evaluation policy. Suppose the treatment and control groups do not satisfy the parallel trend assumption. In that case, i.e., there are specific differences before the procedure occurs. The results obtained empirically using the DID model are very likely to have the influence effect of other factors and cannot represent the net effect of the policy (41).

This paper draws on the research methods of Liu (42) and Lyu (43) etc., and generates the dummy variables of the experimental group and time for multiple periods before and after the occurrence of the policy before and after the regression analysis again, and obtains the results of the parallel trend test as shown in Table 7. The results show that the dummy variables representing the period before the policy occurred (pre_3, pre_2, pre_1) are insignificant. In contrast, the dummy variables representing the period after the policy occurred (current, post_3, post_4) are all positively correlated with the explanatory variable IQ at least at the 5% significance level, which indicates that the policy effect is significant. The results show that the trend of the dependent variables in the experimental group and the control group before the occurrence of the policy is the same, thus passing the parallel trend test.

**Table 7:** Parallel trend test results.

Variables	IQ
Pre_3	0.070 (0.94)
Pre_2	0.077 (1.14)
Pre_1	0.111 (1.67)
Current	0.134** (2.39)
Post_1	0.090 (1.68)
Post_2	0.009 (0.21)
Post_3	−0.134** (−2.76)
Post_4	0.328*** (7.94)
Size	−0.038 (−0.65)
Lev	0.084 (0.78)
Cash	0.023* (2.14)
Roe	0.183* (1.88)
Growth	0.001 (1.56)
Shareh10	0.001 (0.97)
Comp	−2.902*** (−4.59)
Constant	1.590 (1.30)
Observations	1,507
R-squared	0.429
Company	Yes
Year	Yes

Robust *t*-statistics in parentheses. *** *p* < 0.01, ** *p* < 0.05, * *p* < 0.1.

### 5.5 Robustness checks

A series of robustness tests are required to ensure the credibility of the results of the Difference-in-Differences model. Drawing on existing literature, the main robustness tests used in this paper exclude other events interference methods, the placebo test, and the PSM-DID test.

#### 5.5.1 Exclusion of other events

The outbreak of COVID-19 in 2020 had a significant impact on the global public health system and Chinese pharmaceutical manufacturing firms. Statistically, this serious public health event is a strictly exogenous external shock to firms’ operations, and the time interval of the sample selected in the paper contains 2020 and 2021, which, if not considered, may lead to serious endogeneity problems in the empirical analysis due to the omission of variables. Therefore, this paper draws on the research methods of An (44) and Yan (45), then excludes the unique samples in 2020 and beyond and conducts the regression again. The regression results are shown in Table 8. As can be seen, in this case, regardless of whether the control variables are included, the core explanatory volume significance of the previous conclusions remains consistent, indicating that the results are robust, i.e., the implementation of the policy of consistency evaluation of generic drugs effectively improves the quality of innovation in the pharmaceutical manufacturing industry.

**Table 8:** Robustness test results excluding the effect of COVID-19.

	Excluding the effect of COVID-19	Excluding the effect of COVID-19
Variables	IQ	IQ
DID	0.068*** (6.83)	0.073*** (7.16)
Size		0.025* (1.94)
Lev		−0.102*** (−5.50)
Cash		0.000 (0.03)
Roe		0.138*** (7.78)
Growth		−0.000 (−0.17)
Shareh10		−0.001 (−0.93)
Comp		0.065 (0.49)
Constant	0.638*** (254.10)	−0.367 (−1.31)
Observations	1,123	1,123
R-squared	0.494	0.499
Number of groups	202	202
Company	Yes	Yes
Year	Yes	Yes

*t*-statistics in parentheses. *** *p* < 0.01, ** *p* < 0.05, * *p* < 0.1.

#### 5.5.2 Placebo test

**Figure 2:**
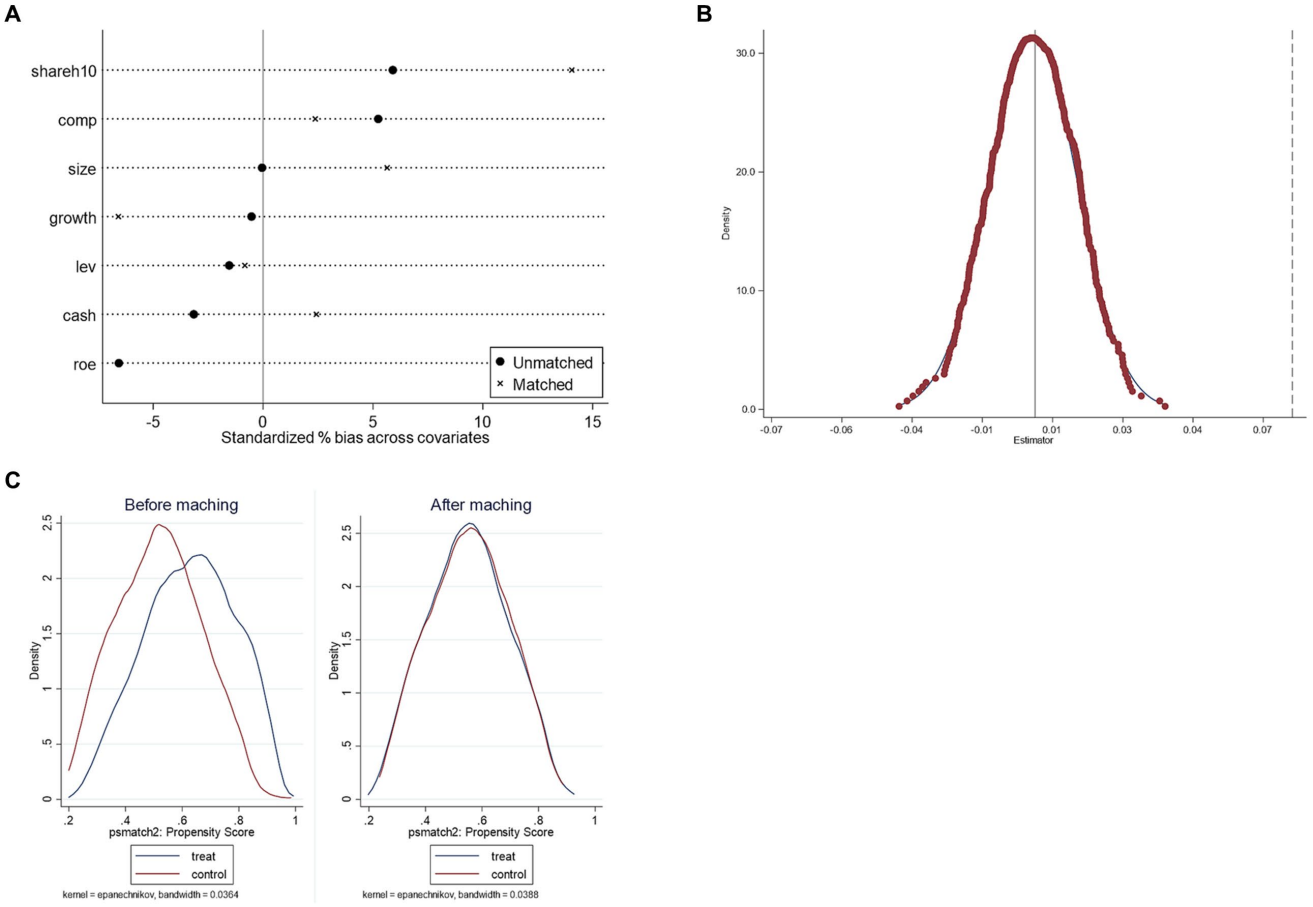
The results of robustness checks. **(A)** The results of the placebo test. **(B)** Standardized deviation before and after matching. **(C)** Propensity score distributions for treatment and control groups.

To reduce the influence of other unobservable potential factors on the relationship between the policy dummy variables and the dependent variable, this paper adopts the counterfactual assumption for the robustness test concerning the research ideas of Shi (41) and La Ferrara et al. (46). Among all the enterprises in the sample, randomly select the same number of enterprises as the original treatment group to construct the “pseudo-treatment group,” the rest of the enterprises as the “control group,” and the “pseudo-treatment group” and test the time dummy variables under the condition that the time point of the policy remains unchanged. Under the state that the time point of the policy remains intact, the “pseudo-treatment group” interacts with the time dummy variable to form a new did variable and then conducts regression again to determine whether the “pseudo-policy dummy variable” coefficient is significant. The software plotted the kernel density of the coefficients after repeating the above operation 500 times, and the results are shown in Figure 2A. The figure shows the distribution of the estimated coefficients of the 500 randomly generated “pseudo-policy dummy variables” and the corresponding density values, where the X-axis indicates the size of the estimated coefficients of the “pseudo-policy dummy variables,” the Y-axis shows the density values and the vertical dotted line is the actual treatment group. The vertical dotted line is the estimated DID coefficient of the treatment group, 0.073. We can see that the distribution of the coefficients of the “pseudo-policy dummy variables” generally follows the normal distribution and is mainly concentrated near the zero point. The actual regression coefficients fall in the domain of a small rejection probability, indicating that the consolation test passes. The results suggest that those unobserved potential factors will not affect the results of the benchmark regression, and the results are robust. Implementing a generic drug consistency evaluation policy can promote the improvement of innovation quality of pharmaceutical manufacturing enterprises.

#### 5.5.3 PSM-DID test

In this paper, seven factors were selected as control variables to be included in the benchmark regression to reduce the impact of factors other than policy implementation on the quality of innovation. Still, the empirically selected combination of control variables will produce a certain degree of selective bias, leading to biased results in the benchmark regression. Using the propensity score matching (PSM) method can effectively mitigate this problem (47, 48), in which individuals in the control group are matched with individuals in the treatment group according to the “proximity” of their respective characteristics (variables in the set of control variables), which results in no significant difference between the matched individuals except for whether or not they receive the policy treatment, which in turn results in a certain level of bias in the baseline regression results. Therefore, this paper combines propensity score matching (PSM) and Difference-in-Differences (DID) and uses PSM-DID to conduct a more profound robustness test, first matching the treatment group with the control group through the PSM method to determine the reduction of self-selection bias and then regressing the samples obtained from the matching to verify the robustness of the baseline regression.

Specifically, this paper adopts the method of 1:1 near-neighbor matching for matching between control variables, with the caliper value limited to 0.01. The changes in the differences of covariates before and after matching are shown in Table 9 and Figure 2B. The differences of all covariates have significantly narrowed by judging the changes before and after bias in Table 9. The *t*-test has changed from the original significant to non-significant; in other words, the covariate similarity between samples has continuously improved, and the sample selection error has alleviated; moreover, by the distribution of the kernel density of the propensity score before and after matching in Figure 2C, the difference between the distribution of propensity scores of the control group and the treatment group after matching significantly reduced, and the trend goes to the same direction, indicating that the data after matching are balanced, i.e., the matching is effective. Finally, the samples obtained after propensity score matching are included in the DID model and regressed again, and the results are shown in Table 10, from which we can see that the core explanatory variable still has a significantly positive coefficient at the 1% significance level, which is consistent with the previous conclusion of the baseline regression, indicating that the results are robust.

**Table 9:** Propensity score matching (PSM) results.

Variable	Matched	Treated	Control	%bias	|bias|	*t*	*p* > |t|	V(C)
Size	U	22.25	21.82	50.40		9.630	0	1.18*
	M	22.25	22.17	8.500	83.20	1.740	0.0830	1.080
Lev	U	0.338	0.269	42.70		8.130	0	1.25*
	M	0.334	0.341	−4.500	89.40	−0.880	0.378	0.86*
Cash	U	1.056	1.425	−24		−4.690	0	0.64*
	M	1.068	1.099	−2	91.70	−0.450	0.652	0.890
Roe	U	0.0783	0.0974	−17.80		−3.440	0.00100	0.890
	M	0.0814	0.0542	25.30	−42.20	4.340	0	0.40*
Growth	U	−0.408	−0.389	−0.500		−0.0900	0.930	1.070
	M	−0.441	−1.226	18.80	−4,005	3.040	0.00200	0.42*
Shareh10	U	56.69	59.28	−17.90		−3.430	0.00100	1.020
	M	56.88	56.92	−0.300	98.40	−0.0600	0.953	0.960
Comp	U	0.165	0.172	−21.30		−4.050	0	1.34*
	M	0.166	0.166	−1.200	94.30	−0.260	0.798	1.22*

**Table 10:** Regression results of PSM-DID.

	(1)	(2)
Variables	IQ	IQ
DID	0.079*** (5.84)	0.092*** (5.92)
Size		0.036*** (2.96)
Lev		0.009 (0.11)
Cash		0.009* (1.93)
Roe		0.337*** (5.63)
Growth		−0.000 (−0.14)
Shareh10		0.002* (1.94)
Comp		−1.605*** (−3.86)
Constant	0.670*** (110.44)	−0.533* (−1.79)
Observations	727	727
R-squared	0.367	0.383
Number of groups	198	198
Company	YES	YES
Year	YES	YES

*t*-statistics in parentheses. *** *p* < 0.01, ** *p* < 0.05, * *p* < 0.1.

### 5.6 Heterogeneity analysis

Since the nature of each enterprise’s equity and the located region is differ, which will lead to different strategic choices and business strategies, as well as the resources and policy support available to the enterprise, it is also necessary to conduct a heterogeneity analysis to ensure the accuracy of the results of the benchmark regression. In this paper, we analyzed the sample firms for enterprise nature and regional heterogeneity according to the different nature of enterprise equity and the located regions.

#### 5.6.1 Shareholding heterogeneity

According to the division criteria provided by the CSMAR database, the sample enterprises are divided into state-owned enterprises and non-state-owned enterprises, respectively included in the baseline regression model for regression after the results are shown in Table 11. The regression results show that the consistency evaluation policy has a more obvious promotion effect on the innovation quality of both state-owned and non-state-owned pharmaceutical manufacturing enterprises. The promotion effect on the innovation quality of non-state-owned enterprises is more significant. The test results of the coefficient of difference between the groups are effective at the 1% level, which further confirms the truth of the theoretical hypothesis H1. The difference between the two, the possible reason, is that, compared with state-owned enterprises, the management system and organizational structure of non-state-owned enterprises are more flexible and flat. Non-state-owned enterprises are susceptible to policies and will pay extra attention to and respond to the state’s policies that directly impact their development.

**Table 11:** Results of the nature of shareholding heterogeneity analysis.

	State-owned	Non-state-owned	Test for difference in coefficients between groups
Variables	IQ	IQ	IQ
DID	0.070*** (4.38)	0.086*** (7.98)	0.083*** (11.77)
DID*Soe			−0.036*** (−4.31)
Soe			0.062 (1.24)
Size	0.189** (2.54)	−0.004 (−0.57)	0.027*** (3.63)
Lev	−0.054 (−0.80)	0.049 (0.63)	0.047 (0.74)
Cash	−0.014** (−2.43)	0.022** (2.45)	0.017* (1.95)
Roe	0.190*** (4.82)	0.113*** (3.91)	0.090*** (4.09)
Growth	−0.000 (−0.39)	0.002*** (2.68)	0.001*** (3.78)
Shareh10	−0.001 (−0.74)	0.000 (0.76)	0.001 (1.56)
Comp	−0.908*** (−2.81)	−1.030 (−1.62)	−1.200* (−1.97)
Constant	−3.919** (−2.49)	0.399* (1.86)	−0.291* (−1.91)
Observations R-squared	339 0.471	1,179 0.321	1,518 0.342
Number of groups	49	195	231
Company	Yes	Yes	Yes
Year	Yes	Yes	Yes

*t*-statistics in parentheses. *** *p* < 0.01, ** *p* < 0.05, * *p* < 0.1.

#### 5.6.2 Regional heterogeneity

According to the located regions, the research object is divided into east, central, and west. It is included in the benchmark regression model for regression results, as shown in Table 12. From the regression results, we can see that the consistency evaluation policy has a more obvious promotion effect on the innovation quality of pharmaceutical manufacturing enterprises in different geographic regions and the promotion effect on the enhancement of innovation quality of the pharmaceutical manufacturing industry in the central area is the highest, followed by the western part, and the eastern region is the smallest. The possible reason is that relative to the east region, the west and central areas of the pharmaceutical manufacturing industry’s development level is relatively low, resulting in its existing innovation quality not being high, and the implementation of a generic drug consistency evaluation policy, a greater degree of stimulation of the central and western relatively backward areas and innovation quality of the enterprise’s innovation and development of consciousness so that the policy of China’s west and central regions of the net effect of the policy is relatively higher than that of the eastern part; and Compared with the western region, the central area has better resource endowment, location advantage, and human resource advantage, therefore, under the same policy impact, it will produce a positive response more quickly than the western region. The theoretical hypothesis H1 is confirmed once again.

**Table 12:** Results of the regional distribution heterogeneity analysis.

	Eastern	Central	Western
Variables	IQ	IQ	IQ
DID	0.063*** (4.96)	0.136*** (11.27)	0.103*** (3.24)
Size	−0.009 (−0.69)	0.038 (1.07)	0.154*** (2.89)
Lev	0.092*** (3.74)	0.386*** (4.46)	−0.430*** (−3.65)
Cash	0.028*** (3.28)	0.056*** (6.69)	−0.032*** (−6.76)
Roe	0.062 (1.62)	0.060 (0.46)	0.141 (1.51)
Growth	−0.000 (−0.06)	0.001 (0.50)	0.001 (0.58)
Shareh10	0.000 (0.22)	0.004*** (3.03)	−0.003* (−1.91)
Comp	−1.470** (−2.18)	−1.771** (−2.28)	−0.807*** (−3.94)
Constant	0.631** (2.54)	−0.764 (−1.07)	−2.961** (−2.43)
Observations	921	322	275
R-squared	0.328	0.410	0.462
Number of groups	144	46	41
Company	Yes	Yes	Yes
Year	Yes	Yes	Yes

*t*-statistics in parentheses. *** *p* < 0.01, ** *p* < 0.05, * *p* < 0.1.

## 6 Further analysis: the mechanism of the impact of generic drug consistency evaluation policy on the quality of innovation in the pharmaceutical manufacturing industry

The results of the previous empirical study proved that generic drug consistency evaluation significantly improves the quality of innovation in China’s pharmaceutical manufacturing industry, so how does the policy improve the quality of invention? What is its inherent mechanism? According to the theoretical analysis in the previous section, the procedure can affect the innovation quality of the pharmaceutical manufacturing industry by enhancing the intensity of R&D capital investment (H2) and R&D personnel investment (H3) of enterprises. This part will verify whether this mechanism is valid through empirical evidence.

### 6.1 Equation design

Due to the unavoidable endogeneity problem of the traditional three-step mediation effect test, concerning Liu et al. (49), Wu et al. (50), and Li (51), this paper chooses the two-step method to construct the following model to test the mechanism of the impact of the generic consistency evaluation policy on the quality of innovation in the pharmaceutical manufacturing industry:


(2) $$M_{\mathrm{it}}=\alpha_{\mathrm{it}}+\beta {\mathrm{DID}}_{\mathrm{it}}+\delta {\mathrm{Control}}_{\mathrm{it}}+\varepsilon_{\mathrm{it}}$$

Where ${\mathrm{DID}}_{\mathrm{it}}$ is a policy dummy variable, obtained by cross-multiplying the dummy variable indicating the treatment group with the dummy variable indicating the time point of policy intervention, which is assigned a value of 1 if enterprise i participated in the generic consistency evaluation at time point t, and 0 otherwise. $M_{\mathrm{it}}$ is a mediating variable, i.e., the intensity of research and development (R&D) capital investment (RDI) and research and development (R&D) personnel investment (RDP); and ${\mathrm{control}}_{\mathrm{it}}$ is a combination of control variables. The test results are shown in Table 13. It can be seen that the implementation of generic drug consistency evaluation policy has a significant positive effect on the improvement of both R&D human input intensity and R&D capital input intensity of enterprises.

**Table 13:** Results of mechanism tests.

	(1)	(2)
Variables	RDP	RDI
DID	0.567** (2.30)	1.065*** (3.10)
Size	0.659*** (3.32)	−0.136 (−1.31)
Lev	−2.728*** (−4.94)	−1.307 (−1.37)
Cash	−0.425*** (−3.69)	−0.118 (−1.27)
Roe	−3.915*** (−4.69)	−9.503*** (−6.58)
Growth	−0.028*** (−3.32)	0.067*** (4.53)
Shareh10	−0.034** (−2.23)	0.016* (1.68)
Comp	4.621 (1.23)	2.858 (1.65)
Constant	1.649 (0.48)	9.589*** (4.09)
Observations	1,518	1,518
R-squared	0.618	0.160
Number of groups	231	231
Company	Yes	Yes
Year	Yes	Yes

*t*-statistics in parentheses. *** *p* < 0.01, ** *p* < 0.05, * *p* < 0.1.

Existing studies generally agree that increased R&D capital investment intensity has a significant positive effect on innovation quality (52–54). According to Wernerfelt’s “The Resource-Based Theory of the Firm,” on the one hand, the increase in R&D investment by enterprises provides reliable funding, personnel, and access to information for innovation activities, promotes the development of new products, and enhances the innovative capacity and quality of enterprises (55). On the other hand, continuous R&D investment will form a cumulative effect, strengthen the ability of enterprises to capture and evaluate frontier technologies promptly and facilitate enterprises to learn, imitate, and absorb frontier technologies promptly, which indirectly improves the benefits of enterprises’ innovation output, and then motivates enterprises’ willingness to increase the intensity of R&D investment further to form a benign cycle (56). From the perspective of the nature of innovation activities, according to Schumpeter’s point of view (57), innovation is the establishment of a new production function, the introduction into the production system of a combination of production factors and production conditions that have never existed before. It is the whole process for the first time that research and development results have been commercialized and applied. Therefore, high-quality innovation is highly dependent on in-depth research in basic science. The increase in R&D capital investment allows enterprises to have more resources to conduct primary research, which in turn serves to enhance the ability to digest and absorb technology and provide a long-term knowledge reserve for the realization of breakthrough innovations, thus contributing to the enhancement of the quality of innovation in enterprises.

In addition, the essence of innovation-driven development is talent-driven development. The study points out that maximizing the subjective initiative of talents is an essential reason for developed countries to be able to walk at the forefront of the world (58); Koroglu et al.’s (59) study shows that advanced human capital is a crucial resource for enterprises to achieve a competitive advantage, and a higher level of human capital implies a higher learning ability, which can effectively promote innovative activities. Feldman and Audretsch (60), based on the economic and social development data of 97 regions in Germany, found that the increase in the size of R&D talent is one of the critical factors in promoting the level of regional innovation quality. Feldman and Audretsch (60) believes that when scientific and technological skills form a particular scale, it will not only significantly improve the quantity of scientific and technological innovation but also produce a leap in the quality of innovation. Zhao (61) and other researchers pointed out that high-intensity R & D personnel investment so that enterprises have a more vital knowledge creation ability, can continue to explore and accumulate to achieve breakthroughs in core technology, have a more critical ability to digest and absorb, through the transformation of the introduction of technology, to realize the second innovation. Moreover, high-level R&D personnel have strong externalities, which can enhance the knowledge level of middle- and low-skilled laborers through education and training and promote the generation of high-quality innovation. Thus, the increase in the investment intensity of R&D personnel in enterprises will facilitate the improvement of enterprise innovation quality.

According to social psychology, individual decision-making will be affected by the decision-making of other individuals in a specific group, thus showing a tendency to converge with other individuals in the group regarding behavior, a phenomenon known as the cohort effect (62). Combined with the research of Song et al. (63), both R&D capital investment and R&D personnel investment have the “cohort effect,” i.e., enterprises in the same group will learn from each other and imitate each other in the R&D investment, which in turn can improve the innovation quality of more enterprises and the whole industry. Therefore, through the above theoretical analysis, combined with the empirical results of the mechanism test obtained by model (2) (see Table 13), as well as the results of the benchmark regression obtained by model (1) (see Table 6), we can confirmed that the policy of consistency evaluation of generic drugs improves the quality of innovation in China’s pharmaceutical manufacturing industry through the enhancement of the intensity of the R&D capital investment and R&D human resources investment in the pharmaceutical enterprises in this path. So far, hypotheses H2 and H3 are confirmed.

## 7 Conclusions and implications

The generic consistency evaluation policy is not only a core reform objective of China’s drug review and approval system reform but also a critical link in the implementation of the three doctors’ linkage and the advancement of medical and healthcare system reform, and an essential means to promote the structural adjustment of China’s pharmaceutical manufacturing industry and improve the international competitiveness of China’s pharmaceutical products. In this paper, the implementation of a generic drug consistency evaluation policy is regarded as a quasi-natural experiment, and the impact of this policy on the innovation quality of the pharmaceutical manufacturing industry and its mechanism of action is empirically analyzed using the double-difference method. The following conclusions are obtained: (1) The policy has a significant positive impact on the innovation quality of China’s pharmaceutical manufacturing industry, and the robustness of the empirical results is verified by excluding the effect of the new crown epidemic, utilizing the PSM-DID method and the placebo test method, respectively. (2) This paper analyzes the heterogeneity of the sample enterprise grouping based on the nature of equity and geographic region. The results show that whether it is a state-owned enterprise or a non-state-owned enterprise, and regardless of whether the enterprise is located in the East, Central, or West, the implementation of the policy of consistency evaluation of generic medicines can promote the enhancement of the quality of innovation in the pharmaceutical manufacturing industry. (3) This study combines empirical and theoretical analyzes to investigate the influence mechanism of the generic consistency evaluation policy to enhance the innovation quality of the pharmaceutical manufacturing industry and finds that the policy enhances the innovation quality of the entire pharmaceutical manufacturing industry by enhancing the intensity of R&D capital investment and R&D personnel investment of pharmaceutical manufacturing enterprises. Based on the above research findings, the following insights are gained from this paper:

1. Firmly and regularly implement the policy of consistent evaluation of generic drugs. Implementing generic drug consistency evaluation is not only upgrading existing generic drugs and product quality improvement but also qualitatively upgrading China’s pharmaceutical manufacturing innovation system and innovation quality. Therefore, it is necessary to firmly implement the policy of consistently evaluating generic drugs and promote the high-quality and normalized implementation of this policy at all levels of regulatory units and enterprises.
2. The goal of implementing the consistency evaluation of generic drugs is to improve the quality of generic drug products and narrow the clinical efficacy gap with the original drugs. Still, there is more to implement this policy. Generic is the basis of innovation, and innovation is the premise of generic. Therefore, the generic consistency evaluation policy should be regarded as an indirect innovation incentive policy to continuously improve the quality of independent innovation in China’s pharmaceutical manufacturing industry and drive the development of domestic first generic drugs and even original drugs.
3. Emphasize the unified implementation and resource deployment of generic drug consistency evaluation policy in different regions. As the consistency evaluation policy does not have regional heterogeneity in enhancing the quality of innovation in the pharmaceutical manufacturing industry, it is essential to focus on inter-regional coordination and harmonization in the implementation of the policy and also take into full consideration the different geographic locations, resource endowments and pharmaceutical manufacturing capabilities of each province in China, and give appropriate resource tilts and policy support to enterprises with poor geographic locations, poorer resources, and weaker capabilities.
4. Utilize practical policy tools to assist the high-quality implementation of generic drug consistency evaluation policy. The empirical results show that the generic drug consistency evaluation policy affects the quality of innovation by improving the intensity of R&D capital investment and R&D human resources investment. Therefore, formulating support policies related to R&D capital investment and personnel investment aspects can further enhance the effect of generic drug consistency evaluation on innovation quality. For example, provide financial support to enterprises purchasing reference preparations through government subsidies or enterprise tax rebates, or high-level R&D personnel can be attracted to join pharmaceutical enterprises by formulating policies encouraging the settlement of research personnel.

Compared with existing studies, this paper provides evidence of empirical research for the generic consistency evaluation policy to promote the pharmaceutical manufacturing industry to improve the quality of innovation. It provides an in-depth analysis of the policy’s impact from the impact mechanism’s perspective, which enriches the research results on the consistency evaluation policy of generic drugs. However, this study still has certain limitations. First, due to the restrictions of the data source, this paper only empirically analyzes the listed pharmaceutical manufacturing enterprises, resulting in a small sample size included in the benchmark regression, which may have a particular impact on the conclusions; second, this paper only analyzes the mechanism from the intensity of R&D capital investment and R&D personnel investment, and in practice, there may be other factors as mediating variables that have an impact on the quality of innovation, resulting in this paper not being comprehensive enough to analyze the mechanism. Thus, the mechanism analysis in this paper needs to be more in-depth. Therefore, in future research, it is necessary to collect better sample data and conduct a comprehensive and in-depth analysis of more mechanism factors (e.g., the degree of market competition, the degree of financing constraints, government subsidies, etc.) affecting the quality of innovation.

## Data availability statement

The original contributions presented in the study are included in the article/supplementary material, further inquiries can be directed to the corresponding authors.

## Author contributions

QL: Conceptualization, Data curation, Formal analysis, Software, Writing – original draft. ZH: Methodology, Supervision, Writing – review & editing. ZM: Methodology, Supervision, Validation, Writing – review & editing.
